# Using Methods From Computational Decision-making to Predict Nonadherence to Fitness Goals: Protocol for an Observational Study

**DOI:** 10.2196/29758

**Published:** 2021-11-26

**Authors:** Marie McCarthy, Lili Zhang, Greta Monacelli, Tomas Ward

**Affiliations:** 1 Insight Centre For Data Analytics Dublin City University Dublin Ireland

**Keywords:** decision-making games, computational psychology, fitness goals, advanced analytics, mobile app, computational modeling, fitness tracker, mobile phone

## Abstract

**Background:**

Can methods from computational models of decision-making be used to build a predictive model to identify individuals most likely to be nonadherent to personal fitness goals? Such a model may have significant value in the global battle against obesity. Despite growing awareness of the impact of physical inactivity on human health, sedentary behavior is increasingly linked to premature death in the developed world. The annual impact of sedentary behavior is significant, causing an estimated 2 million deaths. From a global perspective, sedentary behavior is one of the 10 leading causes of mortality and morbidity. Annually, considerable funding and countless public health initiatives are applied to promote physical fitness, with little impact on sustained behavioral change. Predictive models developed from multimodal methodologies combining data from decision-making tasks with contextual insights and objective physical activity data could be used to identify those most likely to abandon their fitness goals. This has the potential to enable development of more targeted support to ensure that those who embark on fitness programs are successful.

**Objective:**

The aim of this study is to determine whether it is possible to use decision-making tasks such as the Iowa Gambling Task to help determine those most likely to abandon their fitness goals. Predictive models built using methods from computational models of decision-making, combining objective data from a fitness tracker with personality traits and modeling from decision-making games delivered via a mobile app, will be used to ascertain whether a predictive algorithm can identify digital personae most likely to be nonadherent to self-determined exercise goals. If it is possible to phenotype these individuals, it may be possible to tailor initiatives to support these individuals to continue exercising.

**Methods:**

This is a siteless study design based on a *bring your own device* model. A total of 200 healthy adults who are novice exercisers and own a Fitbit (Fitbit Inc) physical activity tracker will be recruited via social media for this study. Participants will provide consent via the study app, which they will download from the Google Play store (Alphabet Inc) or Apple App Store (Apple Inc). They will also provide consent to share their Fitbit data. Necessary demographic information concerning age and sex will be collected as part of the recruitment process. Over 12 months, the scheduled study assessments will be pushed to the subjects to complete. The Iowa Gambling Task will be administered via a web app shared via a URL.

**Results:**

Ethics approval was received from Dublin City University in December 2020. At manuscript submission, study recruitment was pending. The expected results will be published in 2022.

**Conclusions:**

It is hoped that the study results will support the development of a predictive model and the study design will inform future research approaches.

**Trial Registration:**

ClinicalTrials.gov NCT04783298; https://clinicaltrials.gov/ct2/show/NCT04783298

## Introduction

### Background

As a society, we are becoming more sedentary. In some countries, inactivity levels can be as high as 70%, with 1 in 4 adults and 3 in 4 adolescents not achieving the recommended World Health Organization (WHO) activity levels [1]. Recommendations for adults are as follows: “at least 150 minutes of moderate-intensity aerobic physical activity throughout the week or do at least 75 minutes of vigorous-intensity aerobic physical activity throughout the week or an equivalent combination of moderate- and vigorous-intensity activity” [2]. In a press release issued to celebrate world health data on April 7, 2020, the WHO stated that approximately 2 million deaths per year are due to low physical activity. From a global perspective, sedentary behavior is one of the 10 leading causes of mortality and morbidity [1]. Physical inactivity can significantly affect an individual’s health and quality of life and increase the probability of developing several chronic diseases such as heart disease, obesity, high blood cholesterol, and type 2 diabetes.

Every year, a large section of the global population makes resolutions that are focused on improved fitness. Unfortunately, these resolutions are not sustained, with both popular media and peer-reviewed journals reporting on the low number that maintain behavioral changes. Popular media report dramatic figures, including an analysis by Strava of their global membership data, revealing that January 17, 2020, was *quitters day*, the day individuals are most likely to give up their New Year’s resolutions [3]. Similar statistics have been reported for gym membership and use. A review by Sperandei et al [4] of over 5240 gym members revealed a dropout rate of 47% by the second month, 86% by the sixth month, and 96% by the 12th month. These figures were reinforced by a Spanish study of 14,522 gym members, which revealed that between 47.3% and 56% dropped out over the course of a year [5].

Nevertheless, despite the health impacts and our good intentions, the questions remain: why do so many start but abandon their fitness regimens? What factors impact this nonadherence to self-made fitness goals, and can we use computational models of decision-making to predict those most likely to give up? Does the issue reside in the resolution itself and tied to that specific goal-setting activity? Is it down to motivation, behavioral change, and personality traits, or is it a complex combination of the aforementioned factors? This study’s unique value is the combination of computational psychology methods, personality traits that have been shown to correlate with *healthy behaviors*, and behavioral change with objective physical measurement using a fitness tracker. The study hypothesis hopes to show that using a decision-making game, combined with contextual insights from personality traits, a predictive behavioral model can be built that can be used to identify individuals most likely to give up personal physical activity goals. The ability to predict a specific phenotype based on results from the decision-making tasks, Type D personality, and self-efficacy will allow targeted motivational health interventions. These personality traits will be described in greater detail in the following sections.

### Goal-Setting

Goal-setting is seen as an essential factor in commencing and sustaining health behaviors [6]. A significant body of work focuses on goal-setting as a strategy for promoting sustained physical activity. The best-known and most widely implemented is the goal-setting theory developed by Locke and Latham [7], which is based on the premise that human behavior is purposefully regulated by an individual’s goal. The successful outcome of sustained healthy behavioral change is impacted by the individual constructs of goal-setting theory, such as effort toward goal-related activities, persistence, and commitment [8]. In a recent reflection, Locke and Latham reviewed 50 years of the development of the goal-setting theory and reaffirmed that the approach has “generality across participants, tasks, nationality, goal source, settings, experimental designs, outcome variables, levels of analysis, and time spans” [9]. However, the current school of thought has further refined this approach and dissected goal-setting into two separate domains of performance goals and learning goals, with the former more appropriate for those who are already physically active and the latter more impactful on novices or those new to physical activity [10].

### Self-efficacy

Self-efficacy has been defined as *the belief in one’s capabilities to organize and execute the courses of action required to manage prospective situations* [11]. There has been a considerable interest in the impact of low self-efficacy on human behavior, particularly its effects on physical activity levels [12]. A 2012 review by Bauman et al [13] explored 5 separate categories, which are demographic or biological, psychosocial, behavioral, social and cultural, and environmental factors, and identified a positive correlation between self-efficacy and physical activity. Self-efficacy has been identified as a core belief that affects each of the basic *processes of personal change.* Bandura further described how individuals with low self-efficacy were more likely to give up [14]. He developed a targeted questionnaire to measure self-efficacy associated with reduced physical activity, the 5-item Self-Efficacy for Physical Activity [15].

### Type D Personality

A Type D personality is a term used to describe a personality type that tends to have negative affectivity and social inhibition [16]. Denollet [17] developed the DS14, a validated psychometric measure for assessing negative affectivity, social inhibition, and Type D personality types, and as early as 1998, it showed a demonstrable link between Type D personality and coronary heart disease. According to a 2005 study of over 3800 subjects, this 14-item questionnaire was observed to be stable over 3 months and not dependent on mood and health status [16]. Type D personality has been associated with medication nonadherence and heart failure [18], coronary heart disease [19], and type 2 diabetes [20,21]. Several studies have linked Type D personalities with sedentary lifestyles, including a 2009 study of 564 healthy men that showed that Type D personality was more common in men with a sedentary lifestyle (45%) than men who exercised regularly (14%) [20]. Compared with non-Type D personality types, Type D personality types have been associated with decreased walking and total exercise [22]. This study of 189 healthy volunteers looked at the relationship between Type D personality, physical activity, and self-efficacy and determined that Type D personalities had lower levels of self-efficacy and engaged in significantly less walking and total exercise compared with non-Type D personalities [22].

Low self-efficacy has been associated with Type D personalities in populations with chronic diseases such as type 2 diabetes [23] and acute coronary syndrome [19].

### Status of Change

When exploring physical activity, particularly those individuals who are embarking on a new exercise program, understanding an individual’s perspective concerning their status of change will provide critical contextual insights. The current understanding regarding behavior change suggests that individuals need to progress through several changes. The most widely used model is the status of change model, which forms part of the 10-stage transtheoretical model [24]. The status of change model consists of 5 separate stages: *“*(1) precontemplation where no intention to change is intended within the next 6 months, (2) contemplation where change is intended sometime in the future (usually defined as between 1 and 6 months), (3) preparation where change is intended in the immediate future (1 month) and steps are taken to help prepare for change, (4) action where the target behavior has been modified for <6 months, and (5) maintenance that is the stage characterized by temporally robust behavior change extending beyond 6 months” [25]. Individuals are believed to transition progressively through the various stages. They can regress to the previous stages. Perhaps it is not surprising that self-efficacy, a situation-specific construct [26], is believed to change as the individual moves through the status of change [15,25]. We selected the WHO physical activity recommendations as a guideline for this study [27].

### Computational Psychology and Decision-making Games

There has been a growing interest in applying computational modeling to understand human behavior. The ability to predict human behavior and understand that drivers behind the decision-making process have significant application in adherence to physical activity behavior and relevance when predicting medication adherence in health care. Decision-making tasks have evolved within computational psychology to simulate real-life decision-making and sensitivity to reward and punishment. As described by Ahn et al [28], the performance of simulated gambling tasks represents a conglomerate of psychological processes such as reinforcement learning and motivational strategies. One of the best-known games is the Iowa Gambling Task (IGT), developed by Bechara et al in 1994 [29], and has been used extensively to evaluate the decision-making process. This assessment uses 4 decks of cards labeled A, B, C, and D. Two decks are good, and 2 are bad. If the good decks are selected, there are positive outcomes. If the bad decks are selected, there are corresponding negative outcomes. Throughout the game, the player learns to select the positive decks.

In a study by Bechara, a discernible difference in deck selection was identified when performance of healthy participants was compared with that of a clinical population. Although initially, the IGT was used in subjects with damage to the ventromedial prefrontal cortex, it has been used in a broader range of clinical patients and healthy populations to detect risky decision-making. Within the clinical context, the IGT has been used in several populations where decision-making is impaired, such as gambling, substance abuse, and several neurological conditions such as schizophrenia and psychopathy [30] and eating disorders [31]. Regarding healthy populations, some work has been done to show a differential response depending on whether individuals self-select as either an intuitive (affective) decision-making style or deliberate (planned) decision-making style, potentially those with a more intuitive decision-making style having greater success on the IGT [32]. Research in the '80s and '90s suggested a possible link between negative mood and decision-making behavior [33,34]. Suhr et al [35] built on these findings and investigated the impact personality traits have on the IGT’s performance. In 2007, they published the research results on 87 nonclinical participants, which showed that higher negative mood correlated with risky performance on the IGT. In 2013, the team identified a correlation among mood, personality variables, and deck selection. Although this study did not identify a link between positivity and a specific deck selection, less advantageous decks were selected by individuals in a more negative mood [36]. Although not without distractors, these findings were supported by the Somatic Marker Hypothesis, which postulates that emotional defects significantly impact what is understood as the normal decision-making process [37]. The hypothesis also specifies the number of structures and operations required for the normal decision-making operations.

Although this is one of the best-known decision-making games, some limitations have been reported, with some researchers reporting both interstudy and interindividual variability in healthy participants. It has been proposed that the IGT lacks *an integrated sensitivity measure* [38]. Other contradictory findings have been reported, which are against the premise that healthy subjects learn to select decks that yield the highest returns, the good expected value decks. Several studies have shown that participants select the less favorable decks with larger penalties and that there may be a drive to choose more frequent gains and immediacy of reward rather than long-term outcomes. This is referred to as *gain-loss frequency* [39,40] and healthy participants are influenced by the frequency of losses rather than the long-term outcomes [41]. In 2013, Steingroever et al [41] suggested that performance inconsistencies impacted the value of IGT to measure real-life decision-making. However, in their aptly named paper, “Who Fails the Iowa Gambling Test (IGT)?” Suhr et al [42] ascribed several plausible explanations to the IGT *failed performance* in normal populations, including personality and negative mood.

Several groups have developed strategies to overcome the limitations of the IGT. There have been numerous evolutions of decision-making games, such as the Soochow Gambling task [43]. This is a more symmetrically designed game, with a more defined expected value between the good and bad decks. Participants were offered only a single net payoff for each trial. According to the team that developed the Soochow Gambling Task, *gain-loss frequency rather than expected values guide decision-makers,* this desire for instant gain may explain some impulsive behaviors in real life [43].

Many researchers have focused on modifying the empirical cognitive modeling used to identify meaningful signals to characterize the underlying human behavior behind the tasks’ choices. The established Reinforcement Learning model includes the Expectancy-Valence Learning model [44] and the Prospect Valence Learning (PVL) model [45]. Modifications include PVL-Delta [46], PVL-DecayRI [47], PVL2 [48], and the Value-Plus-Perseverance model [49]. In 2018, Haines [50] proposed a model called the Outcome-Representation Learning model that provided the best compromise among competing models [50]. This model has been tested in a healthy nonclinical population and will be one of the leading models used in this study. Steingroever [51] proposed other novel Bayesian analyses. To date, most of the models have been trained and tested in clinical subjects, with few applied to normal nonclinical populations.

Decision-making games based on game-theoretical ideas, such as the Prisoner’s dilemma game and the Trust game, which are reciprocal games involving social decision-making, were deemed to be outside the scope of this study.

### Study Hypothesis

The hypothesis of this study is as follows: combining results from decision-making tasks, such as the IGT with contextual insights gleaned from personality trait assessments and objective data from the physical activity tracker, we can develop a predictive model that can identify those most likely to give up their fitness goals. This information has the potential to be used to create more targeted support to ensure that those who embark on fitness programs are successful. This study also acts as a feasibility model to test the deployment of questionnaires and the IGT using a *bring your own device* (BYOD) model. The intention is to incorporate feedback from compliance and acceptance testing to inform future studies.

## Methods

### Study Design

This multimodal observational study combines objective sensor data with decision-making games and contextual personality traits to identify patterns in exercise decay. The data generated will build computational models to predict digital personas, who are most likely to abandon exercise goals.

### Recruitment

The study will be advertised on social media, commencing in 2021. This is an entirely virtual, BYOD study design, and interested individuals without any underlying health issues and over the age of 18 years will be invited to download the study app from the Google Play store (Alphabet Inc) and Apple App Store (Apple Inc). Those who are willing to consent to the study and own a Fitbit (Fitbit Inc) will be requested to participate in the study. Once consent is given to participate in the study, participants will be asked to share their Fitbit data. Physical activity, sleep, and heart rate data will be shared using an anonymized token system to ensure that only pseudonymized data are included in the study. Once the individuals provide consent to participate in the study and share their Fitbit data, they will be asked to complete the following assessments: demographics, Type D personality, goal-setting, and self-efficacy questionnaire. They will also be asked to perform decision-making games based on the IGT.

Metrics concerning compliance with each assessment will be quantified. Data will be captured over 12 months. At the end of the study period, participants will be sent a notification to thank them for their participation and a link to where the study results will be shared once available.

### Research Participants

Healthy adults (self-certified) over the age of 18 years who are embarking on a physical activity regime will be invited to participate in the study. These subjects need to own their own Fitbit and smartphone and be willing to participate in the study.

### Study Interventions

#### Study App

The study app was developed by Dublin City University (AthenaCX, DCU). It allows researchers to rapidly design and deploy mobile experience sampling apps (iOS and Android), including integrated consenting and wearable data collection devices. This will provide the participants with a study overview in the form of plain language statements and privacy statements and facilitate the participants e-consent into the study. The app was designed to ensure that subjects have read the study details and consented to the study before completing the study assessments (Multimedia Appendix 1 includes a text sample from the plain language statement and informed consent in the mobile app). The participants have the right to withdraw from the study at any time. The app will be used to deliver the assessments and provide a link to the decision-making game. Push notifications will be sent to the participants during the study, requesting them to complete the assessments at the required time intervals, as detailed below.

#### Fitbit

The use of fitness trackers in this study will facilitate the objective assessment of the participants’ activity patterns and behavioral changes in exercise. It will also enable the identification of individuals who already have a well-established exercise regime. A BYOD approach will be used, and data from each participant’s Fitbit will be captured. Fitbit devices are a range of physical activity trackers and smartwatches that combine integrated accelerometers and photoplethysmograph sensors to measure motion and heart rate. Proprietary algorithms convert the accelerometer data into sleep and activity patterns. Fitbit devices use Bluetooth low energy technology to synchronize with the individual’s mobile device and produce various metrics, including step count, floors climbed, distance covered, calories burned, active minutes, sleep time and stages, and heart rate. In this study, a high-frequency intraday data will be collected using a web-based application programming interface. These data are highly granular and include 1 minute and 15 minutes for activity and 1 second and 1 minute for heart rate.

### Assessments

#### Demographic Information

Participants will be requested to provide details of their age and sex. The following classifiers will be used: male, female, self-defined, and prefer not to say.

#### Self-efficacy Questionnaire

The self-efficacy questionnaire for exercise was based on the Bandura Exercise Self-Efficacy scale initially developed in 2006 [26], and the original 0 to 100 numerical rating scale was used (Multimedia Appendix 1). Participants were asked to rate their degree of confidence by moving a widget across a scale on the app. A rating of 0 equates to *cannot do at all* and a rating of 100 reflects complete confidence and participants are *highly certain* they can engage in physical activity. Participants will be requested to complete the self-efficacy questionnaire at the start of the study and 6 months after the study commencement (Multimedia Appendix 2 includes the self-efficacy questionnaire and screenshots of the app).

#### Status of Change

To ensure that all participants were able to assess their current activity levels against the same criteria, the WHO physical activity guidance [27] was included in the instructions for the *stages of change* questionnaire. Participants were asked to consider only planned physical activities aimed at improving or maintaining physical fitness and health. Active periods should consist of 150 minutes of moderate-intensity physical activity (such as a brisk walk) in a week or 75 minutes of vigorous-intensity physical activity (such as a jog or run) over the course of a week. Participants will be asked to complete the status of changes questionnaire at the start of the study and 6 months after study commencement (Multimedia Appendix 3 includes details of the stages of change questionnaire and screenshots of the app).

#### Type D Personality Questionnaire

The 14-question Type D personality questionnaire developed by Denollet [16] will be used in this study. A total of 7 questions pertain to the negative affectivity domain and 7 to the social inhibition domain. Each question will be scored on a scale of 0 to 4. The questionnaire will be scored as individual negative affectivity and social inhibition domains and a composite Type D personality score. Participants will be asked to complete the Type D personality questionnaire at the start of the study and 6 months after the study commencement (Multimedia Appendix 4 includes details of the Type D personality questionnaire and screenshots of the app).

#### Decision-making Tasks

These tasks will be performed twice during the study. The IGT will be deployed within the first 3 months and at 6 months. Participants in the IGT will be given €2000 (US $2314) virtual money and presented with 4 decks of cards labeled A, B, C, and D. Each card in these decks can generate wins and can sometimes cause losses. Participants must choose 1 card from these 4 decks consecutively until the task shuts off automatically after 100 trials. In each trial, feedback on rewards and losses of their choice and the running tally over all trials so far are given to the participants, but no information is given regarding how many trials they will play and how many trials they have completed during the task. Participants will be instructed to choose cards from any deck and switch decks at any time. They will also be told to make as much money as possible, minimizing losses.

**Figure 1:**
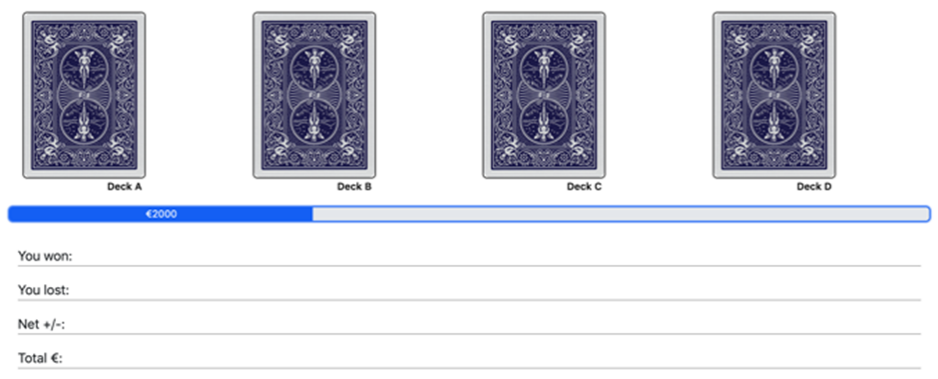
Screenshot of the web-based Iowa Gambling Task.

Table 1 presents the payoff for the 4 decks. As seen in the table, decks A and B are 2 “bad decks” that generate high immediate, constant rewards but even higher unpredictable, occasional losses. Thus, the long-term net outcome associated with decks A and B is negative. In contrast, decks C and D are 2 “good decks” that generate low immediate, constant rewards but even lower unpredictable, occasional losses. Thus, the long-term net outcome associated with decks C and D is positive. In addition to the payoff magnitudes, the 4 decks also differ in the frequency of losses, that is, decks A and C are associated with a higher frequency of losses, whereas decks B and D are associated with a lower frequency of losses. The key to obtaining a higher long-term net outcome in this task is to explore all the decks in the initial stage and then exploit the 2 good decks (Figure 1 shows a screenshot of the web-based IGT implemented).

**Table 1:** Summary of the payoff of the Iowa Gambling Task.

Characteristics	Deck A (bad deck with frequent losses)	Deck B (bad deck with infrequent losses)	Deck C (good deck with frequent losses)	Deck D (good deck with infrequent losses)
Reward/trial	100	100	50	50
Number of losses/10 cards	5	1	5	1
Loss/10 cards	−1250	−1250	−250	−250
Net outcome/10 cards	−250	−250	250	250

### Data Analysis and Computational Modeling

This study aims to investigate whether we can predict adherence to self-made fitness goals and identify potential behavioral phenotypes based on the changes in the participants’ responses to a series of validated instruments, including self-efficiency, status of change, and Type D personality, and their behavioral performance on the IGT over 6 months. A range of data analysis techniques will be applied to the collected data to achieve this goal.

We term the results obtained from questionnaires and decision-making tasks as predictor variables, whereas the adherence levels of the participants to fitness goals as target variables for convenience of description. The first problem that must be addressed before predicting and clustering is to quantify both the predictor variables and target variables.

Self-efficiency was measured by an instrument including 18 items using the original 0 to 100 numerical rating scale, where 0 represents *cannot do at all* and 100 represents *high certainty to do.* The Type D personality questionnaire also uses numerical rating scales, although with different ranges from 0 representing *false* to 4 representing *true*. However, for the Status of Change questionnaire, the answers were categorical scales of *yes* or *no*. Thus, the one-hot encoding technique, one of the most common encoding methods in machine learning, will convert the categorical answers to numerical formats.

To evaluate participants’ performance on the IGT, both superficial behavioral variables and underlying cognitive parameters will be summarized and estimated from the behavioral data set. Specifically, 2 parameters will be measured in the behavioral summary analysis. The first parameter will be the total amount of gain by the end of the task, and this parameter will be used to measure the overall performance of the IGT. The second parameter will be the IGT learning scores across the task, that is, the difference between the number of good deck selections and that of bad deck selections across the task, used to reflect the learning process in the task. The 100 trial choices will first be divided into five blocks of 20 trials, each without overlap, and the learning score will be calculated for each block. As a result, 5 variables (learning scores) will be created for this measure.

Cognitive parameters that drive the behavioral performance will be extracted from the cognitive models designed for the IGT. As mentioned earlier, multiple models have been proposed for the IGT, including the Expectancy-Valence Learning model, the PVL-Delta model, the PVL-Decay model, the Value-Plus-Perseverance model, and the latest proposed Outcome-Representation Learning model (Table 2 presents the parameter specifications of the 4 IGT models). The hierarchical Bayesian modeling method, where both individual and group parameters (ie, posterior distributions) are estimated simultaneously in a mutually constraining fashion, will be used to estimate the cognitive parameters for each model. The models will be implemented in a newly developed probabilistic programing language STAN, which uses a specific Markov Chain Monte Carlo sampler called Hamiltonian Monte Carlo, to efficiently perform sampling from high dimensional posterior distributions as specified by the user. After obtaining the fitting results, model validation will be executed to determine the winning model, including a one-step-ahead leave-one-out information criterion and a posterior predictive check. The parameters of the winning model will then be used as predictor variables to predict adherence behavior. Thus, the number of variables obtained here for predicting adherence depends on which model best fits the data set.

**Table 2:** Parameter specifications of the 4 Iowa Gambling Task models.

Model	NP^a^	Parameters							
PVL^b^_delta	4	Outcome sensitivity (α)	Loss aversion (λ)	Learning rate (*A*)	Response consistency (c)	N/A^c^	N/A	N/A	N/A
PVL_decay	4	Outcome sensitivity (α)	Loss aversion (λ)	Decay parameter (*A*)	Response consistency (c)	N/A	N/A	N/A	N/A
VPP^d^	8	Outcome sensitivity (α)	Loss aversion (λ)	Learning rate (*A*)	Decay parameter (*K*)	Gain impact parameter (*EP*_P_)	Loss impact parameter (*EP*_N_)	Weight parameter (*w*)	Response consistency (c)
ORL^e^	5	Reward learning rate (A_+_)	Punishment learning rate (A_–_)	Decay parameter (*K*)	Outcome frequency weight (*ß*_F_)	Perseverance weight (*ß*_p_)	N/A	N/A	N/A

^a^NP: number of parameters.

^b^PVL: Prospect Valence Learning.

^c^N/A: not applicable.

^d^VPP: Value-Plus-Perseverance.

^e^ORL: Outcome-Representation Learning.

Finally, the question arises of how to quantify the target variable, that is, the adherence behavior, given that various types of objective fitness data will be collected. We will mainly rely on the data set of activity patterns and the step counts. According to the WHO physical activity guidelines, to improve or maintain physical fitness and health, active periods should consist of 150 minutes of moderate-intensity brisk walking or 75 minutes of vigorous-intensity jogging or running over the course of a week. If the participant completes this minimum exercise in a particular week, their adherence score for this week will be 1; otherwise, the score is 0. The scores for all weeks will be summed to obtain a total score to represent the overall adherence level of this participant.

### Making Predictions and Clustering

It is apparent that the numerical predictor variables are measured at different scales; therefore, a standardization process is necessary to make the numerical features identical regarding the range. *Z* score normalization that scales the value while considering the SD will be adopted in this case. Finally, a list of regression algorithms in machine learning will be applied to the cleaned data set, in which we characterize each participant with a vector of a certain length. The possible algorithms include linear regression, rigid regression, lasso regression, and neural network regression.

In addition, unsupervised learning algorithms that can work independently to discover patterns and information will be applied to the data set to identify potential behavioral phenotypes. The K-means clustering algorithm, one of the simplest unsupervised learning algorithms, can be used to solve this problem.

### Risks

Participation in this study presents no identifiable risk to the participants.

## Results

This study was funded by the Irish Research Council as part of an employment-based PhD scholarship. Ethics approval was obtained from Dublin City University in December 2020. At manuscript submission, study recruitment was pending. The expected results will be published in 2022.

## Discussion

### Overview

The rationale for this study is to determine whether data from decision-making games and personality traits could predict individuals most likely to give up on self-determined physical activity goals. The ability to identify the digital persona of those most likely to abandon exercise goals has significant value to those involved in developing targeted support to encourage and adhere to fitness goals.

### Methodological Limitations

#### Recruitment

Participants will not be incentivized to join the study, and as such, it may be challenging to reach the recruitment goals. A rolling strategy will be adopted with regular push notifications to social media and a dedicated study page established on Facebook to maximize recruitment. These pages will have details of the study, the app, and a YouTube instructional video.

#### Loss of Fitbit Data

All Fitbit devices have a finite internal memory, and intraday data are stored for 5 to 7 days, depending on the tracker (daily totals are generally retained for 30 days). If participants do not regularly synchronize their devices, the data can be overwritten.

Participants may fail to complete the required number of tests in the IGT to facilitate parameter modeling and derive the required insights.

Depending on the number of participants recruited for the study, the application of deep learning approaches may be limited owing to the participant numbers and may result in overfitting.

## Appendix

Multimedia Appendix 1 Text sample from the plain language statement and informed consent in the mobile app.

Multimedia Appendix 2 Self-efficacy questionnaire.

Multimedia Appendix 3 Status of change.

Multimedia Appendix 4 Type D personality questionnaire.

## Abbreviations

BYOD: bring your own device
IGT: Iowa Gambling Task
PVL: Prospect Valence Learning
WHO: World Health Organization
