# Supplementary figures and images for: Using Methods From Computational Decision-making to Predict Nonadherence to Fitness Goals: Protocol for an Observational Study
Source: JMIR Res Protoc. 2021 Nov 26;10(11):e29758. doi: 10.2196/29758 (PMC8665389; doi:10.2196/29758)

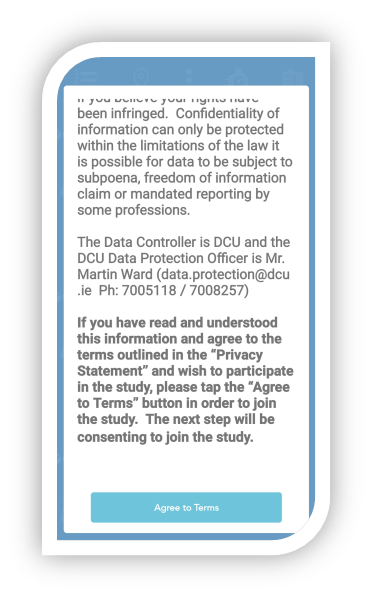

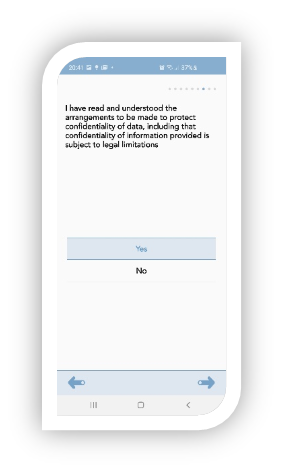

Supplement: Multimedia Appendix 1 [file resprot_v10i11e29758_app1.docx]
