# Supplementary material for: Using Methods From Computational Decision-making to Predict Nonadherence to Fitness Goals: Protocol for an Observational Study
Source: JMIR Res Protoc. 2021 Nov 26;10(11):e29758. doi: 10.2196/29758 (PMC8665389; doi:10.2196/29758)

Rate your degree of confidence by recording a number from 0 to 100 using the scale given below:

0 10 20 30 40 50 60 70 80 90 100


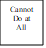


Highly Certainly can do

| A number of situations are described below that can make it hard to stick to an exercise routine. Please rate in each of the blanks in the column how certain you are that you can get yourself to perform your exercise routine regularly (three or more times a week). | Confidence  (0-100) |
| --- | --- |
| When I am feeling tired |  |
| When I am feeling under pressure from work |  |
| During bad weather |  |
| After recovering from an injury that caused me to stop exercising |  |
| During or after experiencing personal problems |  |
| When I am feeling depressed |  |
| When I am feeling anxious |  |
| After recovering from an illness that caused me to stop exercising |  |
| When I feel physical discomfort when I exercise |  |
| After a vacation |  |
| When I have too much work to do at home |  |
| When visitors are present |  |
| When there are other interesting things to do |  |
| If I don't reach my exercise goals |  |
| Without support from my family or friends |  |
| During a vacation |  |
| When I have other time commitments |  |
| After experiencing family problems |  |

Samples of Screenshots


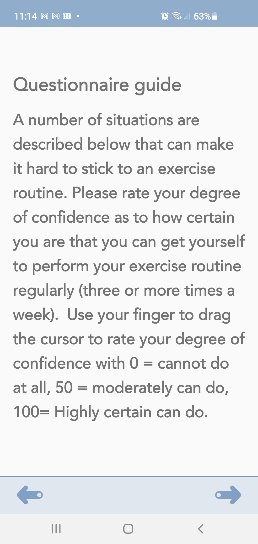

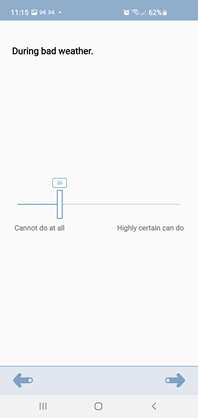

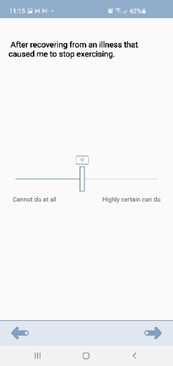

Supplement: Multimedia Appendix 2 [file resprot_v10i11e29758_app2.docx]
