# Supplementary material for: Using Methods From Computational Decision-making to Predict Nonadherence to Fitness Goals: Protocol for an Observational Study
Source: JMIR Res Protoc. 2021 Nov 26;10(11):e29758. doi: 10.2196/29758 (PMC8665389; doi:10.2196/29758)

Participants are requested to answer each statement either yes or no. Physical activity includes activities such as walking briskly, jogging, bicycling, swimming, or any other similar activity. These activities need to be planned physical activities aimed at improving or maintaining physical fitness and health. This should consist of at least 150 minutes of moderate-intensity physical activity (such as a brisk walk) throughout the week, or 75 minutes of vigorous-intensity physical activity (such as a jog o run) throughout the week, or an equivalent combination of moderate- and vigorous-intensity activity. Moderate intensity activity

| 1 | I am currently not physically active and do not intend to engage in physical activity in the next six months | Yes | No |
| --- | --- | --- | --- |
| 2 | I am currently not physically active, but I am thinking about getting more physically active in the next six months | Yes | No |
| 3 | I currently do some physical activity, but not regularly | Yes | No |
| 4 | I am currently physically active, but have only begun doing so within the last six months | Yes | No |
| 5 | I am currently physically active and have done so for more than six months | Yes | No |

*Screenshots:*


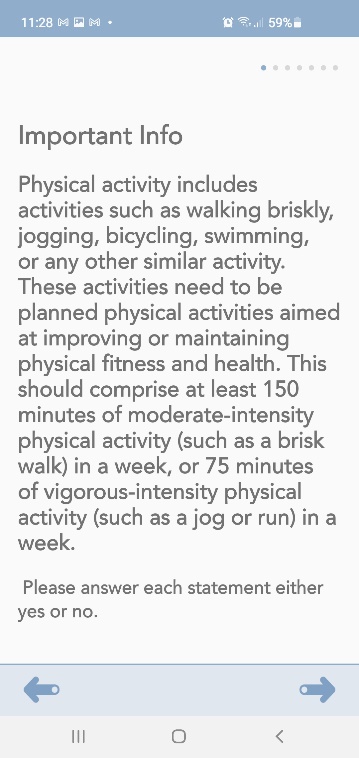

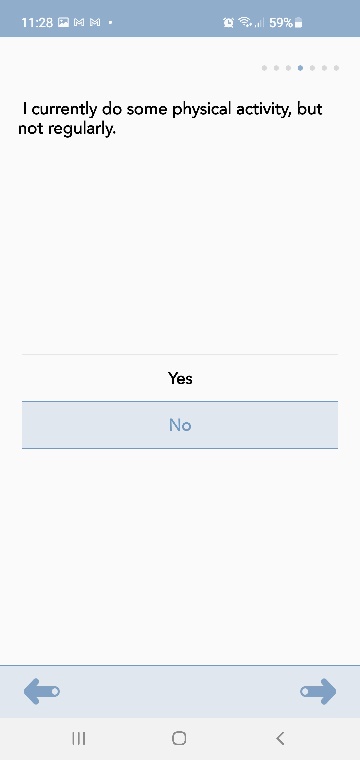

Supplement: Multimedia Appendix 3 [file resprot_v10i11e29758_app3.docx]
