# Supplementary material for: Using Methods From Computational Decision-making to Predict Nonadherence to Fitness Goals: Protocol for an Observational Study
Source: JMIR Res Protoc. 2021 Nov 26;10(11):e29758. doi: 10.2196/29758 (PMC8665389; doi:10.2196/29758)

Below are a number of statements that people often use to describe themselves. Please read each statement and then circle the appropriate number next to that statement to indicate your answer. There are no right or wrong answers: Your own impression is the only thing that matters.

0= False, 1= Rather False 2= Neutral 3= Rather True 4= True

| ^Negative Affectivity Questions^ | | | | | | | ^Social Inhibition Questions^ | | | | | | |
| --- | --- | --- | --- | --- | --- | --- | --- | --- | --- | --- | --- | --- | --- |
| ^1^ | ^I make contact easily when I meet people^ | ^0^ | ^1^ | ^2^ | ^3^ | ^4^ | ^8^ | ^I find it hard to start a conversation^ | ^0^ | ^1^ | ^2^ | ^3^ | ^4^ |
| ^2^ | ^I often make a fuss about unimportant things^ | ^0^ | ^1^ | ^2^ | ^3^ | ^4^ | ^9^ | ^I am often in a bad mood^ | ^0^ | ^1^ | ^2^ | ^3^ | ^4^ |
| ^3^ | ^I often talk to stranger^ | ^0^ | ^1^ | ^2^ | ^3^ | ^4^ | ^10^ | ^I am a closed kind of person^ | ^0^ | ^1^ | ^2^ | ^3^ | ^4^ |
| ^4^ | ^I often feel unhappy^ | ^0^ | ^1^ | ^2^ | ^3^ | ^4^ | ^11^ | ^I would rather keep other people at a distance^ | ^0^ | ^1^ | ^2^ | ^3^ | ^4^ |
| ^5^ | ^I am often irritated^ | ^0^ | ^1^ | ^2^ | ^3^ | ^4^ | ^12^ | ^I often find myself worrying about something^ | ^0^ | ^1^ | ^2^ | ^3^ | ^4^ |
| ^6^ | ^I often feel inhibited in social interactions^ | ^0^ | ^1^ | ^2^ | ^3^ | ^4^ | ^13^ | ^I am often down in the dumps^ | ^0^ | ^1^ | ^2^ | ^3^ | ^4^ |
| ^7^ | ^I take a gloomy view of things^ | ^0^ | ^1^ | ^2^ | ^3^ | ^4^ | ^14^ | ^When socializing, I don't find the right things to talk about^ | ^0^ | ^1^ | ^2^ | ^3^ | ^4^ |

Sample of Screenshots:


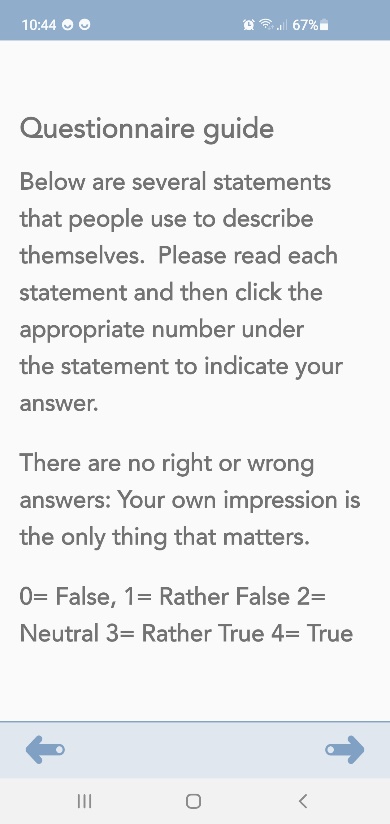

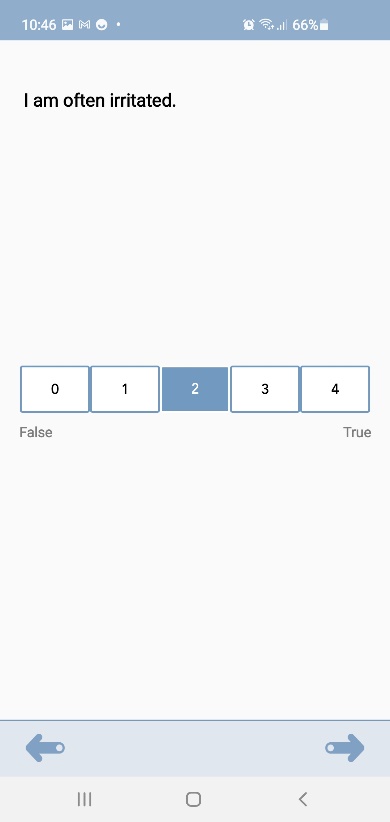

Supplement: Multimedia Appendix 4 [file resprot_v10i11e29758_app4.docx]
